# Annotations

**Published:** 1890-08-09

**Authors:** 


### Annotations.

**Cleanliness and Health.**

"If asked," said Dr. Alfred Hill to the members of the British Medical Association, last week, "what have been the principal agencies by which the past triumphs of preventive medicine have been achieved, there would be little hesitation in answering that the majority of the scourges which have afflicted mankind and been overcome have yielded to cleanliness. . . . The Plague and the mediæval epidemics were banished by cleanliness. Typhus, thanks to the labours of Howard, was influenced by cleanliness as if by magic. Cholera, so fatal in its first visitations before its favouring conditions were understood, wrought terrible havoc where filth conditions prevailed." Dr. Hill reminded his audience of many of those facts and principles which constitute the religion of the sanitarian. Two hundred years ago, he said, the annual death-rate of England was 80 per 1,000 of the population. To-day it is but 20. During the last 17 years only, the death-rate of Salford has diminished 30 per cent. and that of Maidstone 40 per cent. And yet, said the lecturer, there are people in this present year who object to the closure of wells whose water is polluted by sewage, more particularly when the wells are their own. Nothing better proves and illustrates the actual progress of the race of man than the history of sanitation. The intelligent and moral person whose life is spent chiefly among the lowest classes of the people in large towns or villages may well be sympathized with if he sometimes becomes hopeless of any definite improvement either in their morals or their physical condition. But facts like those quoted by Dr. Hill are incontestable proofs that though progress is slow, so slow as almost to be past belief, yet when a large space of time is included within the range of vision, the advancement is as undoubted as it is valuable. That it should have taken so many thousands of years for us to reach our present level, is undoubtedly disheartening. But we must not measure the future by the past. We may reasonably believe that the past has been a prolonged period of preparation, and that now the human intellect, having fairly grasped the principles of a rational science, will move with tenfold rapidity in a forward direction. One part of the business of scientists and sanitarians is to teach; to inform and educate the general mind. Like St. Paul, they should "be instant in season and out of season." Speaking of certain diseases, such as whooping cough and measles, the mortality from which has remained stationary for a long time, Dr. Hill advanced the opinion that "much might be done to diminish the death-rate of these diseases if they were managed in an intelligent manner, and with a genuine desire to cope with them." Perhaps, he said, "this desideratum will not be accomplished until the education of the masses enables them to estimate the actual danger attending these diseases." That, indeed, is the true method of sanitary, as of all kinds of progress. Instruct the people. Teach them to understand intelligently the dangers to which health and life are exposed, and how to remove and to utterly destroy those dangers. The medical profession has been criminally indifferent to its duties in this respect. Thanks to men like Dr. Hill, and other sanitarians, that criminal indifference is beginning to be understood, and the more intelligent of doctors are thoroughly ashamed of it. The public cannot understand the facts and principles of medical science, says the objector. Can they not? Then how do you understand them, sir? Were you not yourself a member of the undistinguished public a few years, or a few score years ago? The truth is, that the public can understand everything the specialist understands, provided it is intelligently taught.

**"Rubbishin."**

"Dr. Smirker is my family physician; he is thoroughly up in all the newest treatment of the times." A most absurd standard to test a man by; and yet the standard adopted by large numbers of the medical profession as well as by the uninstructed general public. Mr. Lawson Tait, one of the most successful specialists of his time, was one of the chief orators in the surgical section at the meeting of the British Medical Association held last week at Birmingham. Mr. Tait gave the members of the association his candid opinion concerning some of the new drugs to which many people are so distressingly ready to pin their faith. "No sooner," said the speaker, "is a new drug placed on the market than everybody rushes to try it. At first all is well, and 'rubbishin' is good for everything. Then come a few isolated hints about the toxic effects of 'rubbishin,' and finally 'rubbishin' gets dropped altogether, and we hear no more about it." This kind of thing is perhaps inevitable in an age of eager scientific inquiry; and it is at any rate much to be preferred to an obstinate prejudice in favour of everything that is established, and a fixed determination to try nothing new. But Mr. Lawson Tait did well to call attention to the subject, and even to hold up the butterflies of medicine to well-deserved ridicule. For if a man be constantly flying after that which is new, it is pretty clear that he has not by prolonged personal experience acquired a firm and reasonable faith in that which is not new. Now there is nothing more sure than this, that certain well-known drugs and familiar methods of treatment in the hands of competent and experienced physicians lead to successful results. Every doctor who is worth the name carries about a trustworthy "Practice of Medicine" in his brain and hands. That which he has proved over and over again he will rely upon with assured confidence. Both for his patient's sake and his own this is what he ought to do. Of course, at suitable times and on suitable occasions and persons, let him try new methods and new drugs. But for a man at the bedside to diagnose a particular disease, and to have in his mind no method of treatment which he has over and over again proved to be reliable, is a very unhappy outlook for his patient. It simply means that every case he treats is the making of an untried experiment so far as he is concerned. Mr. Lawson Tait says the public are largely to blame for the new drug craze, and especially the upper classes. He has been informed by men practising near the dwellings of the princes of the land and at fashionable watering places, that "the burden of their lives is to keep up with the new drugs and the new dodges." Upon which it may be remarked—so much the more contemptible are such doctors for their pains. Is the patient, or are the patient's friends, to teach the doctor how to do his work? Any medical man who makes it his business to suit himself to the whims and fancies of his employers has a dog's life, and he richly deserves it. "For my part," says Mr. Tait, "I instinctively distrust men who are always going in for new drugs."

**Hypnotism and the Daily Press.**

Some of the daily newspapers, whose representatives visited the meetings of the British Medical Association at Birmingham last week, have published leaders on hypnotism, on the ground that it is easy to understand; but they have excused themselves from dwelling on other subjects brought before the Association, because they were so "highly technical." This is truly funny. Of all the subjects discussed by the learned members of the Association, perhaps hypnotism was, on the whole, the most difficult. Anatomy and physiology are highly technical, and are much less familiar to the average reader of the morning paper than are the every-day aspects of medicine and surgery. Yet anatomy is practicality and plain-sailing itself compared with hypnotism. Physiology, though a much more interesting study than anatomy, is yet even more difficult to grasp and thoroughly master. But with physiology is the essence of simplicity compared with hypnotism. If the papers had said that hypnotism was very much talked of just now, was, in fact, the psychical lion of society, as Stanley is the lion of adventure, they would have spoken the thing that is true, and would have offered what would have been generally held as a sufficient excuse for picking out hypnotism instead of other portions of the educational and scientific work in which the members of the British Medical Association are engaged. The truth, indeed, is very evident to all who understand. When the British Medical Association deals with questions of solid and sober science, nobody can spare for it more than a moment's attention. When any of its members enter into competition with conjurers, hypnotisers, and fasting men, all the world rushes to see. The scientific work of the physician, the surgeon, and the physiologist is, to a great extent, boycotted by the general public. This is a very great injury to that work. There are few things that grow so rapidly in a cellar as in the broad light of day. Medicine is not one of them. If the writers of the daily press would take the trouble to understand medical questions two very valuable results would follow: The range of permanently interesting subjects for their columns would be largely increased, and the science and art of surgery would progress with tenfold rapidity under the influence of popular stimulus and criticism.

